# Median Lethal Dose, Antimalarial Activity, Phytochemical Screening and Radical Scavenging of Methanolic *Languas galanga* Rhizome Extract

**DOI:** 10.3390/molecules15118366

**Published:** 2010-11-16

**Authors:** Abdulelah H. Al-Adhroey, Zurainee M. Nor, Hesham M. Al-Mekhlafi, Rohela Mahmud

**Affiliations:** Department of Parasitology, Faculty of Medicine, University of Malaya, 50603, Kuala Lumpur, Malaysia; E-Mails: zuraineemn@um.edu.my (Z.M.N.); halmekhlafi@yahoo.my (H.M.A.); rohela@ummc.edu.my (R.M.)

**Keywords:** *Languas galanga*, methanolic extract, antimalarial activity

## Abstract

The methanolic extract of *Languas galanga* rhizomes was investigated for antimalarial activity against *Plasmodium berghei* (NK65) infections in mice. The median lethal dose was determined to ascertain the safety of the extract in ICR mice of both sexes. The antimalarial activities during early and established infections, as well as the prophylactic activity were evaluated. Phytochemical screening and radical scavenging activity of the extract were also investigated to elucidate the possible mechanism of the antimalarial properties. The acute oral toxicity (LD_50_) of *Languas galanga* extract in mice was established to be 4,998 mg/kg. The extract of *Languas galanga* rhizomes demonstrated significant antiplasmodial activity in all the three models of the antimalarial evaluations. Phytochemical screening revealed the presence of some vital antiplasmodial constituents such as terpenoids and flavonoids. The extract also exhibited a moderate capacity to scavenge the free radicals. The rhizome extract of *Languas galanga* thus possesses antimalarial activity, which explains the rational usage of this plant in traditional Malaysian medicine.

## 1. Introduction

*Languas galanga Syn. Alpinia galanga* (Linn.) Stuntz (Zingiberaceae) is native to grassland areas of Southeast Asia and Southern China [1]. In Malaysia, the rhizomes (Langkuas) of this plant are used as a spice for flavouring food. Ethnobotanically, the rhizomes are used to treat a variety of sicknesses including coughs, headache, asthma, bronchitis, inflammation, rheumatoid arthritis and colic [2,3]. Previous biochemical studies on *Languas galanga* have shown that the rhizomes contain essential antimicrobial components [4,5,6,7,8]. The rhizomes exhibit many pharmacological properties, including antitumour [9], antiallergic [10], antiulcer [11], antifungal [12], antibacterial [13] and antiviral activities [14].

Currently, multi-drug resistance has become one of the most important problems impeding malaria control efforts [15,16]. This has led to attempts to discover other antimalarial agents, mainly from plant sources. Medicinal plants may provide antimalarial drugs directly, as in the case of quinine from cinchona bark, or they may supply template molecules on which to base further new structures by organic synthesis–artemisinin from *Artemisia annua*. The selection of candidate plants for clinical trials depends largely on ethnopharmacological information, which has appeared to be more predictive compared to the random screening approach [17].

In view of the reported antitrypanosomal [18], antileishmanial [19] and antihelmithic [20] antiparasitic effects of *Languas galanga* rhizomes, in the present study we evaluated the *in vivo* antimalarial activity of the methanolic extract of *Languas galanga* rhizomes against early, established and residual malaria infections. Median lethal dose, phytochemical screening and radical scavenging were also studied to ascertain the validity of its folkloric standing.

## 2. Results and Discussion

### 2.1. Median lethal dose

The mortality rate and the acute toxicity symptoms of orally administered *Languas galanga* rhizome extract increased as the dose increased from 2,000 to 5,000 mg/kg (Table 1). The main observed behavioural signs of toxicity were asthenia, piloerection, ataxia, anorexia, urination, diarrhea, lethargy and coma. Asthenia, piloerection and urination were noticed after dosing with 2,000 mg/kg and were more marked at the highest dose and continued until death, in particular among the male subjects, which showed a lethal median dose lower than that of the females. The acute oral toxicity (LD_50_) of *Languas galanga* extract in mice was calculated by the probit analysis to be 4,998 mg/kg. According to Horn [21] and Rhiouani *et al*. [22], plants or plant products with LD_50_ values higher than 2,000–3,000 mg/kg are considered free of any toxicity. This supports the logical usage of this plant in folk medicine practices.

All the treated mice were carefully observed for 14 days for any signs of toxicity (behavioural changes and mortality). D/T: dead/treated mice; none: no toxic symptoms were recorded during the observation period; latency: time to death (in hours) after the dose administration.

**Table 1:** Acute oral toxicity of the methanolic extract of *Languas galanga* rhizomes administered orally to mice.

Dose (mg/kg)	Mortality		Toxic symptoms
	D/T	Latency (h)	
0	0/10	-	None
300	0/10	-	None
2,000	1/10	>36, <60	Asthenia, piloerection, anorexia, urination, diarrhea
5,000	5/10	>24, <60	Asthenia, piloerection, ataxia, anorexia, urination, diarrhea, lethargy, coma

### 2.2. Antimalarial activity

During early malaria infection, the rhizomes extract of *Languas galanga* produced a dose-dependent chemosuppression activity at the different doses employed. 4-day suppressive effects of 29, 49, 63 and 65% were shown, respectively, for the corresponding dose of the extract (50, 100, 200 and 400 mg/kg/day). The antimalarial activity produced by the extract was statistically significant (*P* < 0.05) when related to control (Table 2). However, the 400 mg/kg demonstrated only a marginal increase in activity over 200 mg/kg, suggesting that further increases in the dose produced only marginal increases in activity [23].

**Table 2:** The antimalarial activities of the methanolic extract of *Languas galanga* rhizomes during early, established and residual malaria infections.

Drug/extract	Dose	Parasitaemia	%Chemo-suppression	Significance
**Suppressive activity**				
Control (dist. water)	0.2 mL	5.10 ± 0.33		
Rhizome extract	50 mg/kg	3.60 ± 0.68	29	ns
	100 mg/kg	2.60 ± 0.37	49	*P* < 0.05
	200 mg/kg	1.91 ± 0.91	63	*P* < 0.01
	400 mg/kg	1.81 ± 0.89	65	*P* < 0.05
Chloroquine	20 mg/kg	0	100	
**Curative activity**				
Control (dist. water)	0.2 mL	9.60 ± 0.93		
Rhizome extract	50 mg/kg	5.80 ± 1.21	40	ns
	100 mg/kg	3.40 ± 0.22	65	*P* < 0.05
	200 mg/kg	3.40 ± 0.78	65	*P* < 0.05
	400 mg/kg	3.20 ± 0.77	67	*P* < 0.001
Chloroquine	20 mg/kg	0	100	
**Prophylactic activity**				
Control (dist. water)	0.2 mL	4.60 ± 0.51		
Rhizome extract	50 mg/kg	4.00 ± 0.55	13	ns
	100 mg/kg	3.40 ± 0.93	26	ns
	200 mg/kg	2.80 ± 0.49	39	*P* < 0.01
	400 mg/kg	2.20 ± 0.37	52	*P* < 0.001
Pyrimethamine	1.2 mg/kg	1.24 ± 0.47	73	*P* < 0.05

In the established malaria infection, the plant extract exhibited dose-dependent curative antimalarial activity, which was statistically significant (*P* < 0.05) when compared to control. The mean parasitaemia for the treated groups on the sixth day of infection were 5.80, 3.40, 3.40 and 3.20 for 50, 100, 200, and 400 mg/kg/day, respectively. A marginal increase in activity over 100 mg/kg was produced by the 200 and 400 mg/kg doses. The mean parasitaemia for the control group was 9.60 (Table 2).

As summarized in Table 2, the results of prophylactic activity of the rhizomes extract during the residual malaria infection showed dose-dependent chemosuppression at 50, 100, 200 and 400 mg/kg/day dose levels, exerting 13, 26, 39 and 52% suppressions, respectively. It showed that though there was a significant prophylactic activity (*P* < 0.05) at the 200 and 400 mg/kg doses, the chemosuppressive activity was rather low and not significant at the 100 and 50 mg/kg doses. The rhizomes extract of *Languas galanga* exhibited some level of chemosuppression of parasitaemia. The 100 and 200 mg/kg appeared to be effective therapeutic doses in the early and curative tests. On the other hand, the suppression of parasitaemia by chloroquine at 20 mg/kg/day appeared to be in agreement with a previous study conducted on the chloroquine efficacy in *Plasmodium berghei* NK65-infected ICR mice [24].

The results of the anti-malarial screening assays of the test extract, standard drugs and control groups at different doses in mice parasitized with *P. berghei* are expressed as percentage (%) for the suppression of parasitaemia. ns: non-significant (P > 0.05); *P* values ≤ 0.05 were considered to be statistically significant (effective). Parasitaemia are expressed as mean ± S.E.M. (n = 5). Significance compared to control.

### 2.3. Phytochemical screening

Phytochemical screening of the methanolic extract of *Languas galanga* revealed that the rhizomes extract contains terpenoids, flavonoids, tannins, saponins, steroids and glycosides (Table 3).

**Table 3:** Phytochemical profile of the methanolic extract of *Languas galanga* rhizomes.

Chemical constituents	Tests/Reagents	Results
Alkaloids	Dragendorff’s Reagent	-
Anthraquinones	Sulphuric acid-chloroform layer test	-
Flavonoids	Ammonium Test	++
Glycosides	Fehling’s reagent	+
Saponins	Frothing Test	++
Steroids	Salkowski’s test	++
Tannins	Lead sub-acetate test	+++
Terpenoids	Salkowski’s test	++

+++ = abundance; ++ = moderately present; + = present; **-** = absent

Phytochemical compounds such as terpenoids are commonly implicated in the antiprotozal and antiplasmodial activity of many plants [25,26,27,28]. An example of common terpenoids is artemisinin, the main active ingredient in the traditional Chinese antimalarial qinghaosu. Flavonoids are the other form of *Languas galanga* phenolic structures. Flavonoids showed significant antiparasitic activities against different strains of malaria, trypanosome and leishmania [29,30,31]. These chemical compounds may be acting singly or in synergy with one another to exert the observed antimalarial activity of *Languas galanga*.

### 2.4. Radical scavenging activity

**Figure 1:**
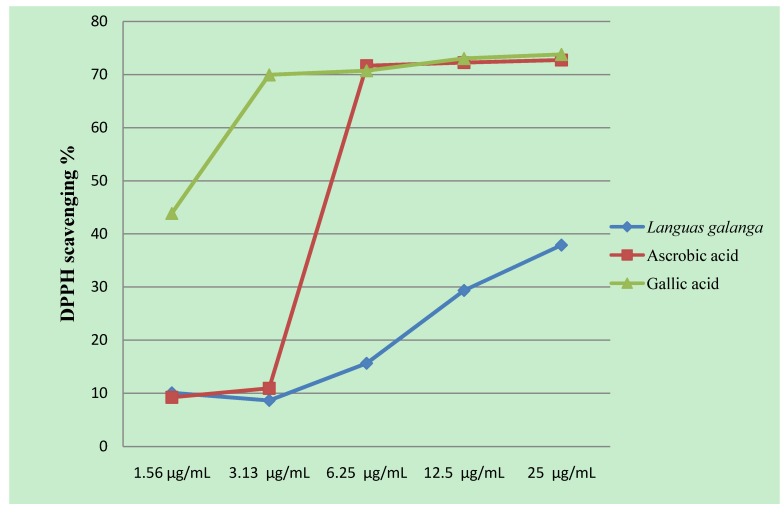
DPPH radical scavenging activities of the methanolic extract of *Languas galanga* rhizomes, ascorbic acid and gallic acid at different concentrations of µg/mL. Absorbance values represent means of triplicates of different concentrations analyzed.

The methanolic rhizome extract of *Languas galanga* showed moderate DPPH radical scavenging activity. At 1.56–25 µg/mL, the scavenging abilities of the methanol extract were 10.08, 8.63, 15.64, 29.34 and 37.88, respectively, as shown in Figure 1. At 25 µg/mL, ascorbic acid and gallic acid showed strong scavenging abilities reaching 72.74 and 73.79%, respectively. The antioxidant effect of the *Languas galanga* rhizomes extract may represent another mechanism that contributes to its anti-malarial activity. Rhizomes extract of *Languas galanga* showed nitric oxide (NO) production inhibitory activities in mouse peritoneal macrophages [32]. NO is a potent intracellular parasite-killing mechanism in macrophages which are crucial in innate immune response. These inhibitory activities assist in providing a favourable environment for the multiplication of intracellular parasites; however, the inhibition of one killing mechanism can cause the up regulation of secondary mechanisms against which the parasite cannot protect itself [33]. The inhibition of NO starves the parasite of an essential amino acid, leading to its death by increasing tryptophan degradation through indolamine deoxygenase induction in human peritoneal macrophages [34].

## 3. Experimental

### 3.1. Plant materials

The plant part (rhizomes) of *Languas galanga* (Linn.) was selected based on our ethnobotanical survey conducted in two malaria endemic communities, forest-aboriginal and rural communities, in the Lipis district of Pahang state, Peninsular Malaysia [35]. The plant has been mentioned as a curative antimalarial remedy by the two communities. The voucher specimen was collected and identified by the University of Malaya Herbarium (KLU) and deposited under the reference number KLU 046619.

### 3.2. Preparation of plant extract

The rhizomes of *Languas galanga* were dried in a hot air oven at 40 °C and milled. Five hundred grams of the powdered materials were soaked in absolute methanol (3.5 L, Merck, Germany) for 72 h. The extracts were concentrated *in vacuo* to dryness at 40 °C using a rotary evaporator and were freeze-dried. The percentage yield was 3.27%. The freeze-dried extract was stored in a refrigerator at −4 °C until used.

### 3.3. Animals

The 6–7 weeks old male (27 ± 2 g) and female (22 ± 2 g) ICR mice that were used for these experiments were obtained from the Laboratory Animal Centre of the Faculty of Medicine, University of Malaya. The mice were housed under standard conditions and were maintained on a standard pelleted feed and water *ad libitum*. Permission and approval for animal studies were obtained from the Animal Ethics Committee of the Faculty of Medicine, University of Malaya dated 05 June 2009 (Ref. No. PAR/05/6/2009/AHAA-R).

### 3.4. Median Lethal Dose

Different doses of the extract (300–5,000 mg/kg) were inoculated to evaluate the acute oral toxicity according to OECD guideline No 423; “Acute oral toxicity – acute toxic class method” [36]. The extract doses were divided into equal parts and given to the animals within a period not exceeding 24 hours. A total of 10 mice (five females and five males) were tested**.** The mice were fasted overnight prior to administration and returned to feeding 3 hrs later. On the day of administration, all the mice were observed for mortality and signs of toxicity at 1, 3 and 4 hrs following administration and thereafter they were observed twice a day for 14 days. The LD_50_ was calculated using probit regression analysis in the SPSS statistical package (version 13, 2004).

### 3.5. Parasite inoculation

The blood of a donor female ICR mouse infected with *Plasmodium berghei* (NK65) was used for inoculum preparation. The desired blood volume was drawn from the donor mouse by heart puncture and diluted serially in Alsever’s solution. The final suspension would contain about 1 × 10^6^ infected RBC’s in every 0.2 mL suspension. This 0.2 mL suspension was injected into mice intraperitoneally to initiate infection [37]. The inoculated animals were then randomized into five mice per cage and maintained in the Animal Room, Department of Parasitology, Faculty of Medicine, University of Malaya, in accordance with the internationally accepted principles for laboratory animal use and care.

### 3.6. In vivo antimalarial assays

A series of experiments were carried out to evaluate the *in vivo* anti-malarial activities of the methanolic extract of *Languas galanga* rhizomes at 50, 100, 200 and 400 mg/kg doses as compared to control groups treated with distilled water (containing 10% DMSO, the solvent of the test extracts) and reference groups treated with standard drugs (chloroquine 20 mg/kg or pyrimethamine 1.2 mg/kg/day). Malaria infection was first established in female mice by the intraperitoneal (i.p.) administration of donor female ICR mouse blood containing about 1 × 10^6^ parasites. The three different methods of treating malaria infections, *i.e.*, 4-Day suppressive test, curative and prophylactic methods were applied according to Peters and Robinson [38], Saidu *et al*. [39] and Peters [40], respectively. The laboratory tests were started with oral administrations of the extracts in the 4-day suppressive tests (early malaria infection) and further screened for their curative (established malaria infection) and prophylactic (residual malaria infection) activities. Thin blood films were prepared from the tail blood of each mouse. The films were then stained with Giemsa’s stain to determine parasitized erythrocytes. The percentage of parasitaemia was determined by counting the parasitized red blood cells out of 9,000 in random fields of the microscope:

% Parasitaemia = [No. of parasitized RBC/Total no. of RBC counted] 100

Average percentage chemosuppression was calculated as: 100 [A-B/A] where, A is the mean percentage parasitaemia in the negative control group and B is the mean percentage parasitaemia in the test group.

### 3.7. Phytochemical screening

The methanolic extract of *Languas galanga* rhizomes was screened for the presence of phytochemical constituents such as alkaloids, terpenoids, anthraquinones, flavonoids, tannins, saponins, steroids and glycosides using qualitative phytochemical screening tests described by Sofowora [41] and Trease [42].

### 3.8. DPPH radical scavenging activity

The radical scavenging activities of the plant extract against 2,2-diphenyl-1-picrylhydrazyl radical were determined photometrically at 515 nm. Radical scavenging activity was measured by using the method of Gerhäuser *et al*. [43]. In brief, DPPH in methanol (195 μM) was added to different concentrations of the extract (5 μL) in a 96-well microplate, incubated for 3 hrs and read at 20-min intervals. Ascorbic acid and gallic acid were used for comparison. The free radical-scavenging activity was expressed as percentage scavenging of the DPPH by the extract and was calculated as follows:

% inhibition = {[Ab-Aa]/Ab} × 100

where Ab is the absorption of the blank sample, and Aa is the absorption of the sample.

Each test was carried out three times and the mean ± SEM were calculated. The DPPH % was presented as μg /mL of concentration.

### 3.9. Statistical analysis

Data obtained by this study were analyzed using SPSS (version 13, 2004). The Student’s t-test and ANOVA (one- or two-way) were used to test the differences between groups. Differences between means at 1 and 5% level (P ≤ 0.01 and 0.05) were considered significant.

## 4. Conclusions

The antimalarial activities shown by the rhizome extract of *Languas galanga* could have been produced as a result of the active antiplasmodial components contained in the extract, mainly terpenoids and/or flavonoids constituents. Antioxidant activity may represent another mechanism that contributes to its anti-malarial activity. Although the bioactive components and mechanism are yet to be identified, the results of this study provide the basis for further studies. This study also confirms the rational usage of this plant in Malaysian medicinal traditions.

## Supplementary Materials

Supplementary File 1
