# Supplementary material for: Median Lethal Dose, Antimalarial Activity, Phytochemical Screening and Radical Scavenging of Methanolic Languas galanga Rhizome Extract
Source: Molecules. 2010 Nov 16;15(11):8366–76. doi: 10.3390/molecules15118366 (PMC6259107; doi:10.3390/molecules15118366)
Supplement: Supplementary File 1 [file molecules-15-08366-s001.pdf]

*Molecules* **2010**, *15*

OPEN ACCESS

*molecules*

**ISSN 1420-3049**

www.mdpi.com/journal/molecules

*Correction*

# Abdulelah H. Al-Adhroey *et al*. Median Lethal Dose, Antimalarial Activity, Phytochemical Screening and Radical Scavenging of Methanolic *Languas galanga* Rhizome Extract. *Molecules*, 2010, *15*, 8366-8376

**Abdulelah H. Al-Adhroey *, Zurainee M. Nor, Hesham M. Al-Mekhlafi and Rohela Mahmud**

Department of Parasitology, Faculty of Medicine, University of Malaya, 50603, Kuala Lumpur, Malaysia; E-Mails: zuraineemn@um.edu.my (Z.M.N.); halmekhlafi@yahoo.my (H.M.A.); rohela@ummc.edu.my (R.M.)

* Author to whom correspondence should be addressed; E-Mail: husssien75@yahoo.com; Tel.: +6-03-79673789; Fax: +6-03-79674754.

*Received: 27 November 2010 / Published: 30 November 2010*

---

The author list given for reference 35 of our paper [1] is incorrect. The revised author list is: Al-Adhroey, A. H.; Zurainee, M.N.; Al-Mekhlafi, H. M.; Rohela, M, so reference 35 should read as follows:

35. Al-Adhroey, A. H.; Zurainee, M.N.; Al-Mekhlafi, H. M.; Rohela, M. Ethnobotanical study on some Malaysian anti-malarial plants: A community based survey. *J. Ethnopharmacol.* **2010**, doi:10.1016/j.jep.2010.08.006.

## Reference

1. Al-Adhroey, A. H.; Nor, Z.M.; Al-Mekhlafi, H.M.; Mahmud, R. Median Lethal Dose, Antimalarial Activity, Phytochemical Screening and Radical Scavenging of Methanolic *Languas galanga* Rhizome Extract. *Molecules* **2010**, *15*, 8366-8376.

© 2010 by the authors; licensee MDPI, Basel, Switzerland. This article is an open access article distributed under the terms and conditions of the Creative Commons Attribution license (http://creativecommons.org/licenses/by/3.0/).
